# Counter acts: practices of ‘anti-anti racism’ in France and the USA

**DOI:** 10.3389/fsoc.2024.1394313

**Published:** 2024-06-21

**Authors:** Steve Garner

**Affiliations:** ^1^Department of Criminology, Sociology and Social Policy, Swansea University, Swansea, United Kingdom; ^2^Faculty of Humanities and Social Sciences, Duy Tan University, Đà Nang, Vietnam

**Keywords:** whiteness, anti-antiracism, state, critical race theory, Islamophobia

## Abstract

In various European countries, the post-fascist nationalist and populist parties identified by Ignazi in the early 1990s ‘silent counter-revolution’ now hold power, at least as part of coalitions. The values they represent can no longer be described as marginal to the national conversations on identity, immigration and security, issues that revolve around racialized understandings of the social world. In recent years we have observed similar phenomena in the Americas and Asia (with the Trump, Bolsonaro and Modi regimes). Moreover, state actors and social movements have developed initiatives aimed at undermining and reversing any small—sometimes symbolic—progress made toward equality. Various attacks on academic concepts relating to racism in the UK, France and the USA, for example, are not isolated stand-alones but elements of a global pushback against such ideas, orchestrated and encouraged by the nationalist political right, working through media, government and funded civil society organisations. These discourses redraw national identity to portray antiracist work as unpatriotic and indeed threatening to the nation. One of the strands in France’s long and fractious conversation about its colonial history and postcolonial present has constructed an opposition between republican values and Muslims. The American right’s long war on racial equality has generated a campaign to eradicate ‘critical race theory’ from education. These two examples illustrate and identify common elements and specifics in a global trend whereby the concepts used by activists and social scientists to understand and frame struggles for racial equality are deliberately and strategically invalidated and vilified in the public domain, and ideologically produced as un-patriotic. I call this discourse ‘anti-anti racism’. These efforts are part of wider campaigns, or ‘counter acts’, aimed at reversing progressive political gains from the last half century.

## Introduction

In the context of trying to understand the contemporary post-Brexit and post-BLM frameworks in which we operate as intellectuals and activists, I identify some contemporary ‘technologies’ (Foucault, 1982) of whiteness. These are discursive practices aimed at neutralizing antiracism as a potential resource for resistance by presenting racism as any or all of the following; obsolete, fictional, irrelevant, a political project aimed at reducing the freedoms of people racialized as white. I suggest how to make sense of a tactic deployed by the State and social movements, whose objective is to counter the advances made in the struggle between racist and antiracist discourses. These counter acts challenge the veracity of concepts developed through activist struggle and academic practice, and undermine their status as valid perspectives. The counter acts push back against concepts accepted by powerful organs of state, which then guarantee discursive normalization, some funding plus political actions, however limited they might be in practice. Moreover, part of the ‘counter act’ involves smearing those who use the concepts in their work or identify with them as rallying points. The current set of exchanges occurs against the backdrop of so-called ‘cancel culture’ in which freedom of speech is harnessed as a means of validating racism and protecting it from critique within the public domain. The argument that freedom of speech is invoked primarily to enable racist speech acts, which then form part of a strategic cycle of victimhood and outrage is rehearsed elsewhere (Mondon and Winter, 2017; Titley, 2020).^1^

This article takes the first step in linking such counteracts as elements of a global pushback rather than a set of national-level phenomena. My reading of the struggles over meaning and power involves drawing connections between these national-level campaigns in terms of content; their engagement with individual nations’ values, and nationalist ideas. Erasing vocabulary, concepts and therefore the discursive basis for organisation and resistance severely damages antiracist social movements’ capacity to function; and where it already exists, detracts from legislation’s function.

First, I will set out the frame for understanding the unusual dynamic of the counteracting: the successful mainstreaming of right nationalist populism in the project referred to by Italian political scientist Pietro Ignazi (1992) as the ‘silent counter revolution’.

## The ‘silent counter-revolution’

Ignazi argued that the development of a Europe-wide movement of post-fascist nationalist populist movements and parties, from the 70s to the early 90s had achieved what he termed a ‘silent counter-revolution’. These groups had mounted feasible and partially successful challenges to Western liberal democratic agendas on immigration, multiculturalism and national independence, identifying national liberal elites as unpatriotic cosmopolitans betraying the national legacies of various European countries by investing more powers in the supra-national European Union, and particularly its cosmopolitan elite leadership, opening borders and experimenting with multiculturalism that allegedly raised crime and lowered wages.^2^

Yet three decades after Ignazi’s paper, that ‘silent counter-revolution’ has become the new establishment in Hungary, and has significantly influenced mainstream political parties in others to the extent that they now share important elements of those originally far-right agendas, even in some Centre-Left parties. Indeed, the counter-revolution Ignazi identified has by now been successful—to various degrees—in most European nations, and with local variations, also in Brazil and India. This counter-revolution contains a significant ideological and discursive aspect, and some of these framings have arguably become hegemonic. However, the two political locations: moving from the margins (1980s) to occupying part of the mainstream (2010s and 2020s), necessitate different types of political strategy for prosecuting the increasingly explicit struggle against anti-racist concepts.

I am going to discuss some of the key strategies deployed—in the ideological realm—to counteract the advances made over recent years in antiracist practice. I understand by ‘advances’ not a linear progression, but an uneven and iterative process of change, whereby legislation, cultural norms and ultimately outcomes become identifiably improved for racialized minority groups. Important advances from this perspective, include the mainstreaming of concepts such as ‘institutional racism’, ‘systemic racism’ and ‘Islamophobia’ *inter alia*, to name specific forms that racism assumes. These advances may largely be symbolic.

This article identifies and analyses two case studies of how the mainstreamed ‘silent counter-revolution’ has generated conflictual engagement with concepts created and utilised by activists and scholars: Critical Race Theory (CRT) in the US, and Islamophobia in France. Some large associated topics therefore lie beyond the scope of this article, namely the broader impacts and implications of right populist nationalist groups, and the panoply of other counter acts aimed at halting and reversing the development of civil rights, equity and diversity, for racialized minorities. Ignazi’s model is invoked specifically to identify that right populist nationalist parties, and more importantly the ideas they operationalize are no longer marginal, although the former might seek to position themselves as counter to the establishment or elites that they relentlessly critique.

Similarly, this is not the story of *all* pushback against equality measures. CRT is just one of several fields of conflict in play since the 1965 Civil Rights Act. Indeed, CRT itself has only existed since the end of the 1980s, and has only been specifically in the firing line for five years. Affirmative Action (Carter and Lippard, 2020), and institutional Diversity, Equity and Inclusion (Moore, 2008, 2018) are others.

### Mainstreaming and populism

By ‘mainstreaming’, I mean absorption into the language of the state and / or civil society, including higher education. Mainstreaming here is ‘contingent’, as posited by Brown et al. (2023), neither referring to fixed boundaries, nor assuming that the mainstream is inherently superior to, or more rational than the margin. Parties and ideas move across landscapes. Moreover, ‘mainstream’ is understood as a cultural space, impacting on the actual practice and ideological shifts that parties and sections of parties effect, rather than being an intrinsic property of ‘mainstream’ parties. For example, the idea that a stringent UK immigration policy is necessary—true for both Conservative and Labour governments (Gilroy, 1987)—can be traced to the 1960s, whereas an emphasis on physical removal and / or encouragement of physical removal of people already in the UK used to be a non-mainstream idea (promulgated by the National Front and the British National Party) until the era of the ‘Hostile Environment policy’ (2013-) and the current administration’s ‘Rwanda policy’. Moreover, the cultural impact of mainstreaming cuts across mainstream parties. A subset of the governing UK Conservative party for instance, has membership of the National Conservatives,^3^ whose statement of principles and list of contributors indicates positions well to the right of that party and beyond.

The deployment of the concept of ‘nation’, both explicit and implicit in the campaigns I am interested in here, is not supposed to indicate that radical populism entails a single and coherent nationalist story. My argument is that elements of the arguments are core to any nationalist group, as much as other elements are vernacular. While right populist nationalist programmes mostly coalesce around security, immigration and anti-elite mobilization, the variation in such elements as the role of religion, the centrality of racialized purity, the economic opposition to or support for welfare spending, for example, should demonstrate that there is no fixed idea of the nation, even among ideological cousins. It would be more accurate to suggest a spectrum of developing options that combine to form a core, which is liable to shift over time.

The current populist project does not actually adopt a centre-left stance on welfare, as has been argued, but an authoritarian version of welfare chauvinism, in which ‘deserving’ groups receive moderate transfers, while ‘undeserving’ ones receive nothing, or are subject to workfare-type policies (Busemeyer et al., 2022). Such imagined welfare regimes can also be deployed as markers of national community, and independence from outside influences deemed inimical to the national interest, as in the case of discourses during the UK’s 2016 EU referendum (Donoghue and Kuisma, 2022). It is exactly the project of careful reworking of discourses within the shell of mainstream constraints into something that shifts the political mainstream that we are interested in here. The French far-right’s adoption of the ‘right to be different’ in the 1980s, and Marine Le Pen’s nationalist, patriotic (and Islamophobic) re-reading of feminism (Shurts, 2024) are examples. The basis of the shift analyzed in this article is the counter act based on the argument that the concepts of ‘race’, racism and anti-racism (plus associated theories and secondary concepts) constitute a divisive force threatening the social coherence of the nation. This strategy enables the public argument that racial discrimination is repugnant and detrimental to the nation, but that liberal racial discourse (i.e., to engage in policy aimed at tackling discrimination, and in conversations about racial discrimination disadvantaging some groups and benefitting others) to divide the nation are equally if not more repugnant.

We will now examine two examples of the state’s^4^ active denial of concepts: ‘Islamophobia’ in France; and the ongoing campaign against ‘Critical Race Theory’ in the USA. These constitute contemporary counter-attacking practices aimed at maintaining white supremacy by delegitimizing critiques of the racial status quo.

So what’s in it for right populist nationalist political projects to resist definitions and concepts used in emancipatory ways? First, igniting fears of disrespect and unfairness (towards people racialized as white) fleshes out the cultural (and affective) strand of a political project now devoid of obvious economic popular selling points. It provides a rallying point that privatizing public assets and lowering taxes for the wealthy cannot galvanize. Additionally, these initiatives delay and distract progressive movements (by making them waste time reiterating the existence of forms of racism), and clear the ideological site for its own doctrines, primarily ‘colourblind racism’ (i.e., individual ‘bad apples’ as opposed to systemic racism; and Islamophobia (in the cases I present below), thus invalidating people’s capacity to link experiences of the past to those of the present (Beaman and Petts, 2020). Lastly, if activists and scholars are unable to effectively use anti-racist concepts in the public domain, the justification for compensatory state action is minimized, and thus the capacity to evoke inequality as an enemy of the commonwealth is reduced.^5^

According to actual CRT (Delgado and Stefancic, 1998) (rather than the version deployed in US discourse), it is only when the interests of elite white groups *converge with* those of minority groups that progressive change can happen *with state sanction*. Therefore, the opposite of this (i.e., when elite white groups’ interests *diverge from* those of minority groups) is implicitly the norm. The timing of the counter acts I am trying to analyse here, tied to global and local events, suggests that is not coincidental, but contingent on a particular configuration of forces, involving the State and social movements with links to the State using common tools to identify concepts and ways of thinking.

I will now turn to the case studies, beginning with the example from France which has a long development, but contains interesting elements that should help us reflect on what power, function and outcomes State-sponsored cancel culture can have.

## Case study 1: Hostility toward the concept of ‘Islamophobia’ in France

Hajjat and Mohammed (2013)^6^ argue that since the early 1980s, the French State has been creating a ‘Muslim problem’, and that the crux of this project entails questioning the legitimacy of the Muslim presence in France, thus presenting Muslims solely as a problem to be solved by control, punishment and / or exclusion from citizenship. Effort is thus exerted in rejecting and invalidating concepts that enable and facilitate critical analysis of the State’s practices.

The ongoing debate about the term ‘Islamophobia’ will probably never be fully resolved. Is it appropriate to bear the suffix ‘phobia’, like an ‘irrational fear’ or a mental illness? Is it really about Islam? Should it be ‘Muslim-phobia’ (Halliday, 1999)? Or is the amalgam between people, beliefs and practices precisely the work that it accomplishes (Meer and Modood, 2019)? My departure point is that there should be some shorthand term we can use to encapsulate a working definition of a form of racism that focuses specifically on Muslims, Islam and people ‘perceived as Muslims’.^7^ It is important to have that so you can name a form of oppression, lobby to have it criminalized and hope for a future where it no longer exists. People who construct an object to protest against and study have chosen the concept of *Islamophobia* as rallying point, and it can be a placeholder to begin a process: particularly if there is state action on the basis of it, such as legislation against such racism. In this case, an imperfect solution is better than no solution.

Notwithstanding its limitations, the concept of ‘Islamophobia’:

1. Helps us recognise that there are deliberate projects carried out by states and social movements, aimed at amalgamating all Islamic practices, Muslims, and people perceived as Muslims, on one hand, with political violence carried out by groups and individuals identifying with Islam, on the other.
2. Explains the process of Muslims being identified as a source of multiple threats to the nation, including organized acts of terrorism and an existential incapacity to assimilate into the norms of the nation.
3. Names a pattern of experiences and structures that can be identified and countered. Numbers 1 and 2 therefore explain the high score of Muslims who in French surveys carried out over recent years, claim to have experienced racist incidents. In 2019, for example, an Ifop / Fondation Jean Jaurès survey (EUObserver, 2019) found that 40% of Muslims had experienced a racist incident in the previous 12 months, a figure that rises steeply to 60% for women who wear hijab. Such experiences are enduring and endemic, and of course not restricted to France (Selod, 2018), but are contributing to an increase in Muslims leaving France (Esteves, 2023). ‘Islamophobia’ enables us to place these experiences within a context and understand their structural, systemic and individual aspects.
4. Enables resistance to be focused and mounted, based on membership of and allyship with the targeted group, regardless of its internal diversity.

So much for what the concept does. The battle I am focusing on here is one in which Islamophobia *as a term* is opposed by many parts of the political spectrum in France, from the Far right to the Far left, and including elements of anti-racist civil society. While the appropriateness of the term is up for discussion, nowhere else does opposition to it generate an alliance of such political breadth (Escafré-Dublet et al., 2023; Zia-Ebrahimi, 2023), and elicit so much hostility from people whose profiles otherwise appear liberal and anti-racist in surveys (Hajjat and Mohammed, 2013: 46–49).

### France as a secular state

Having engaged with the Catholic church as a ferocious defender of the status quo and opponent of republican political values and practices since the late eighteenth century, the leaders of the Third Republic sought to make secularity (*la laïcité*) irreversible, to permanently prevent the Catholic church from significantly interceding again in the world of politics with the 1905 Law on Secularity. *Laïcité* has remained a key value of the Republic: the first lines of the 1958 Constitution state:

‘France is an indivisible, secular, democratic and social Republic. It ensures equality before the law for all its citizens without distinguishing by origins, race or religion’.^8^

This emphasis on official secularity has several important impacts on social inequalities, particularly in terms of measuring and resisting them. Despite the postcolonial migration experienced in France since the 1940s, ways of officially identifying people and groups have not developed along ethnic or racial lines as in the Anglo-American world. Indeed, resisting or denying formal racial categorisation is a constitutive element of French republicanism. Collecting data using such categories is forbidden, and demographers have to produce work-arounds to approximate such exercises (Amiraux and Simon, 2006; Sabbagh and Peer, 2008). While racism is an aspect of French society, political organization based on religion and /or ethnicity / race is largely understood as inappropriate, lying outside the social contract concluded with the triumph of secular Frenchness. Such organisations and identifications are labelled ‘*communautaire*’ (‘ethnically divisive’), and are thus viewed as ‘anti-republican’. While the law on *laïcité* guarantees religious freedom in the non-state (private) domain (the next line of the Constitution is, ‘It respects all beliefs’^9^), it invalidates that freedom in the state (public) domain.

How then does secularity as an official creed impact racism against Muslims and Islam, and efforts to call it out and resist it in the state domain? First, secular France interprets Muslim efforts to prioritize religious identity in their resistance to ‘Islamophobia’ as special pleading because it is based on a religious identity in a state based on secularism. Second, the idea that the concept of ‘Islamophobia’ is used to thwart criticism of Islam and its practices in a secular state is a widely held and frequently recycled affirmation (Bruckner, 2006, 2016; Fourest, 2008; Caldwell, 2009; Ye’or, 2016).^10^ Lastly, Islam is identified—relationally with the liberal, developed practices of France—with backward practices. Thus, political recognition of Islam implied in the acceptance of the existence of the concept and practices covered by the term ‘Islamophobia’ is understood through the secularist republican framing as: condoning restrictions on women’s rights and the abuse of women; an over-intimate relationship between religion and politics that *laïcité* was specifically designed to banish; and barbaric punishments, all of which drag the Republic, and by extension, Europe backwards in time.

### Neo-secularity and the struggle over ‘Islamophobia’

What can we point to as evidence of the State’s development of anti-antiracist tools? Although Hajjat and Mohammed date the focus on Muslim migrants as a threat to the Republic to the strikes in the car-manufacturing industry in the early 1980s, the state’s current engagement with Islamophobia as a concept can be seen on a shorter timeline, beginning with the 2003 Commission on National Identity (the Stasi Commission). This is because the current dominant version of *laïcité* emerged from reflections on that Commission. Hajjat *inter alia*, calls this updated version ‘*neo- laïcité*’ (‘neo secularity’), a term drawn from the title of the position paper of the 2003 Stasi Commission by politician Baroin (2003),^11^ in which he advocates a more assertive and proscriptive version of secularity, specifically to confront the threats represented by Islam in France. Neo-secularity is crucial to understanding the state’s current engagement with both Muslims in France, and concepts deployed to understand that engagement.

In the context of twenty-first century France, the strained ongoing national conversation about colonialism and its legacies (Blanchard, 2006; Bouteldja and Khiari, 2012; Larcher, 2014; Williamson, 2020), relations with the Islamic world, and Islamist political violence in France (particularly since 2015), supporters of neo-secularity seek to create a consensus around the idea of a hierarchy of religious practices. They have re-constructed *laïcité* as the national bulwark against the perceived threat represented by Islam *per se* for French society as a whole, well beyond the very real attacks that have taken place there. Indeed, Islamic culture in general (rather than just people engaged in political violence) is the target for legislation. There are bans on religious clothing (see below), not only in the public sphere, but now within public-sector employment, schools, and private companies carrying out work commissioned by the State. In 2008, the Babyloup creche fired employee Fatima Afif for wearing a headscarf to work, because although it was a private-sector company, it was carrying out a ‘public service mission’ (i.e., nursery education). Afif’s appeal ended up in the French Supreme Court, which ruled in favour of the employer in 2013, establishing a precedent. Criminalizing items of Muslim clothing such as the burqa (2011) and bathing suits (2016) shows the State’s readiness to use secularity as a driver of law, and make Muslims into the frontline of the struggle. Regardless of the consistency with which the laws are actually applied, the key is the idea that the State can regulate cultural aspects out of one designated group in the name of a secular society because it is seen as particularly dangerous, whereas other religious groups are not culturally engaged in such a confrontational way. The regulations also now extend to parents accompanying children on school trips (no conspicuous religious clothing allowed). Just these examples also indicate that the racialization of Muslims is also highly gendered. This can punitive strategy be seen as a continuation of the sporadic and recurrent ‘headscarf affairs’ dating back to the late 1980s, but also a considerable shift in the State’s field of operation. The original state / non-state distinction set out in the 1905 law, is now subject to different interpretations.

Moreover, in 2014, the state repurposed a nineteenth-century law to police publicly-expressed opinions about terrorism.^12^ This law makes it a criminal offence to show support or advocate for terrorism or groups that commit it. Such ‘support’ or ‘advocacy’ is being tested in the courts and has so far included people with mental illness, small children and victims of petty rivalry that lead to allegations being made against them to the authorities. Hundreds of people have been prosecuted using this law so far, and critics have claimed that the State is impinging on people’s freedom of expression by casting the net far wider than actual support and apology for terrorism, to also encompass political comments critical of the French state, for example (Hajjat and Mohammed, 2023), a critique shared by Kundnani (2014) in relation to the UK.

While *laïcité* was originally supposed to protect the right to adhere to religion in the private sphere without impinging on the exercise of citizenship, *neo-laïcité* amalgamates the private and the public, or, expands the public into the private, to the extent where people cannot live out aspects of their culture without cost. Moreover, the creeping criminalization of legitimate democratic disagreement enabled by the application of the apology for terrorism act raises the question of authoritarian intervention into the domain of ideas.

### The neo-secular republic v the concept of Islamophobia

The concept of ‘Islamophobia’ has been a faultline in the struggle to establish neo-secularity. Deploying ‘Islamophobia’ as an analytical concept challenges the one-way traffic of neo-secularity. Through this lens, Islam and Muslims are diverse and plural; there are set of experiences of discrimination shared by Muslims in France, and anti-Muslim racism forms part of a broader set of systemic inequalities that intersect with class and gender. Since it enables an alternative reading of Muslim experiences of the French state, Islamophobia has been identified as an associated threat, and is therefore deliberately linked to support for terrorism and a civic culture antithetical to secular France. As we will see, one of the State’s options is to shoot the messengers, by smearing academics and activists who use Islamophobia as a working concept as enemies of the Republic.

Lastly, the State’s long-standing state disengagement from the concept of ‘race’, viewed disparagingly as an Anglo-American paradigm inappropriate for France (Garner and Fassin, 2013), has entailed not just difficulty measuring demographics or encounters with racism, but also an inability to effectively identify and sanction racism in the public sphere. So while scholars are criticized for using ‘race’, ‘racism’, and ‘Islamophobia’ as working concepts to analyse the social world, surveys carried out by market research companies (rather than academics or activists) repeatedly identify patterns of experiences of racism among Muslims and Black French residents (clearly not completely separate groups) (Richardot, 2023). The state responds to analysis of its actions by using the courts to prevent the use of the term ‘racisme d’etat’ (state racism)^13^ (Gordeau et al., 2020). Indeed, Gordeau et al.’s analysis identifies multiple understandings of race and racism within the discussions on ‘state racism’. With no political consensus on racism, highly politicised debates erupt, with antiracists targeted as problematic, while a thriving counter-culture of racist and islamophobic media has developed in France over the previous two decades. Key players such as Caroline Fourest and Eric Zemmour, as well as a string of high-profile academic experts on Islam and security, such as Rémy Leveau, Gilles Kepel, Bruno Étienne, Bernard Rougier, and Hugues Micheron, who have input into think tanks and policy-making circles, appear frequently in various media (Geisser, 2012). They have generated concepts such as the Islamist ‘ecosystem’, and have developed the concept of ‘separatism’ in terms of describing Muslim activists’ attempts to resist Islamophobia (Hajjat and Mohammed, 2023). Indeed, Zemmour built an entire media persona out of Islamophobia, the ‘great replacement’ theory^14^ and anti-feminism, to the point where he was able to make a run for the Presidency in 2022, occupying space adjacent to Marine Le Pen’s Rassemblement National.

The high-profile academic Pierre-Andre Taguieff coined the terms ‘*islamofascistes*’, and ‘*islamogauchistes*’ (Islamo-Fascists, and Islamo-Leftists), as well as a longer list of terms prefixed by ‘islamo-’. These terms deliberately amalgamate activists and scholars who use the concept of Islamophobia to understand anti-Muslim racism, with groups and individuals that use political violence, to portray them as part of an authoritarian Islamic conspiracy to bring down the Republic. Indeed, Interior Minister Gerard Darmanin referred to such organisations, activists and scholars as ‘enemies of the republic’ (Radio France, 2020). Taguieff and commentators uncritically using his terms are given extensive access to media, which amplifies the neo-secular messages in what are effectively minority voices within academia. In February 2021, Higher Education Minister, Frédérique Vidal demanded that the national research body, the CNRS, conduct an investigation into ‘islamogauchisme’ in French academia (Le Nevé, 2021). Vidal’s request was sternly rejected by the Centre National de Recherche Scientifique, the French national academic research body (CNRS, 2021) on the basis that islamogauchisme is not a valid concept and can therefore not be investigated.

The state’s venture into the realm of policing academia is not in the service of neutrality, but to underscore which perspectives on Islam and race are considered acceptable. As noted above, a number of academics are situated within the State as experts on security and Islam and connected to key media outlets (Hajjat and Mohammed, 2013). According to the anti-islamogauchisme campaigns, American-influenced radical academics, designated ‘fellow travellers’ or ‘useful idiots’ of terrorist groups, dominate academia. The reality is more mundane. There is a tiny number of courses on race and racism in French academia, and a small corpus of scholarly work on these topics. Indeed, according to Patrick Simon and Juliette Galonnier, around 2% of the social science output published in French academia between 1960 and 2020 is about race and racism (Elzas, 2021). The group of scholars using related concepts is relatively small, and more to the point, generally lacking in access to media (Hajjat and Mohammed, 2013). One exception is the independent *médiapart*,^15^ where one can find opinions expressed by scholars working on race, decoloniality, and / or everyday practices of Muslims in France.^16^ In effect, media discourse on Islamophobia is dominated by a very small number of well-known scholars, deploying arguments that identify both Muslims and academics using Islamophobia as a concept hostile to the French Republic.

### State-decreed Islam and the authoritarian turn

To exert control over Muslims in France, and demonstrate its concern for security and national cohesion, the State introduced legislation to collapse the distinctions between Islamic civil society and religious organisations. This key legislation began as the ‘Separatism Bill’ (2020), but eventually had its name changed to “law consolidating respect for the principles of the Republic” (August 2021).^17^ It is worth bearing in mind that historically, the term ‘separatist’ in the French context has been used to describe both Communists (a fifth column) and organisations struggling against colonial rule. The bill was summarised by Minster Jean Castex in a tweet on 9 December 2020 as ‘a supporting freedom. It’s a protective law. A law of emancipation in response to religious fundamentalism’. It enables the Minister of the Interior to close mosques and not-for-profits accused of encouraging and / or supporting terrorist activities. Indeed, hundreds of closures, thousands of investigations and extensive fines resulted from the passage of this law.^18^ This legislation was introduced in December 2020, alongside a Charter, requiring Mosques to sign up to its articles in order to be recognized as officially representing French Muslims by the local authorities, and therefore to stay open.^19^

The most pressing justification of the Bill however was to enable to state to effectively shut down its most vocal critics in the Muslim not-for-profit sector, namely the Collective Against Islamophobia in France (CCIF) and the Islamic Collective against Racism (CICR) (Bechrouri, 2023), under the pretext of their putative links to the October 2020 murder of teacher Samuel Paty. The CCIF in particular had been acknowledged by EU institutions as a dependable partner institution that had been collecting and analysing data on Islamophobia for some time, in a context where such data is rare. Indeed, the French state itself had in one period used the CCIF data. However, in his justification of the anti-Muslim separatism Bill in December 2020, Interior Minister Gérald Darmanin accused the organisation of having “for several years… consistently conducted Islamist propaganda’.^20^ Darmanin also blamed the CCIF for provoking terrorist acts in France by denouncing the state’s anti-terrorism measures as “Islamophobic,” and trying to insinuate that every act committed against Muslims is “Islamophobic” in order to incite public opinion.

The French state’s authoritarian approach to *laïcité* thus represents a mutation of the original concept designed to separate Church and State while enabling religious groups to have access to full citizenship as individuals.^21^ The extension of secularism into the public / non-state domain hosts an ideological campaign aimed at indelibly binding ideas critical of the racialized status quo to terrorism, and casting such resistance and the ensuing political practices it engenders as threats to the Republic. The French state is thus fully engaged in an ideological struggle for which its principal tools are authoritarian updates to legislation.

How do activists and scholars argue and mobilize against something that you struggle to name and cannot collect much data on, because doing so is considered illegal and against the national interest and values, so that when you do engage with the phenomenon it shorthands, you risk being criminalized and have to do so under the state’s definition?

Islamophobia plays an important role in French republican neoliberal order and its recent metamorphosis into more authoritarian culture (Chekkat, 2023; Hajjat and Mohammed, 2023) which makes it all the more crucial for the State to prevent the conversation from becoming a critical one. Nadi (2021) argues that Islamophobia is actually intrinsic to the contemporary configuration of power in France, as it embodies the contradictions of colonial history in the present (what he calls ‘the ideological organisation of neocolonial social relations’ Nadi, 2021: 194). Moreover, the neo-secularity frame successfully fixes Muslims in an exclusively religious identity (i.e., they have no imagined dimensions as actors in any other space) (Hajjat and Mohammed, 2013; Kiwan, 2023). The significance of secularity and the historical resistance to acknowledging the frame of race, and its impact on French universalism combine to make this setting particularly challenging for those struggling to impact the discourse.

## Case study 2: Critical race theory in the USA: the ‘perfect villain’

As of the beginning of January 2024, 30 of the 50 US states either had approved, or were considering enacting legislation that would restrict teaching about race (either as well as, or without mentioning gender): 18 states had bills or similar-level initiatives already passed or signed into law; and 12 had bills passing through state legislature. Another 13 states had bills that had been ‘vetoed, overturned or stalled indefinitely’, while only 7 states had no bill at all.^22^ This constitutes remarkable progress for a movement that only began in Autumn 2019.

### Populist counter acts in the US

The focus here is CRT because it is a concept honed by activists and scholars that is being challenged as part of an anti-anti racism campaign. It is neither *sui generis* nor stands outside a context of reactionary ideas and practices aimed at pushback. There are enduring structures of racism, mitigated only partially by the 1965 Civil Rights Act. The pushback started immediately after that Act, but did not develop momentum until the Reagan administration (Moore, 2018; Embrick et al., 2020), when various activists including current conservative Supreme Court judge, Clarence Thomas got their breaks and attention. Key Reagan advisor Clint Bolick proposed ‘that groups with interest should use politics, media, and courts to attack affirmative action’ (Embrick et al., 2020: 216). Indeed, the campaign against CRT can be understood as one delayed outcome of this broader counter-offensive strategy. Ideas supporting affirmative action, as well as the practice itself have come under legal attack (Harris, 1993). This period essentially saw the start of the grievance politics of white victimization and abandonment that fuel contemporary engagement with DEI for example (Moore, 2008; Embrick et al., 2022).

Cokorinos (2003) argues that the strategy of formal legal challenges, which also structures anti-DEI initiatives, is an elite-generated rather than a grassroots one. He indicates that the vehicles for such a strategy were ‘advocacy groups’ focused on challenging civil rights-related policies, whose numbers increased from around 20 to over 200 in the 1975–1990 period. In the twenty-first century, a set of foundations have lobbied for and funded such initiatives, as well as other relating to women’s reproductive rights and sexuality.^23^ Other individuals have been critical in the journey toward ideological dominance for nationalist populism, such as the Mercer family, who contributed billions of dollars to Trump’s election campaign and to establishing and running Breitbart News (Mirrlees, 2021: 224). It is important to note this because Brown et al. (2023) argue that mainstreaming of populist ideas revolves around the erroneous claim that populists are simply articulating the feelings of the people, rather than creating demand for such solutions by sponsoring mechanisms of top-down racialization of social problems.

The Trump presidency (2016–20) is clearly an essential phase of the story of the rise in American national populism. Trump’s embrace and deployment of racist tropes about immigrants and minorities, his choice of white nationalist Steve Bannon as a senior advisor, his executive order 13769 banning Muslim travellers, his response to the Charlotteville incidents, and his racialization of the Covid-19 pandemic, *inter alia*, normalized what Embrick et al. (2020) label a ‘whitelash’.^24^ In this article I do address forms of ‘whitelash’, and this term fits perfectly in the US context. However, I do not want to include everything outside North America, such as Chinese and Indian state racism against Muslims (Selod et al., 2024) under that heading, which is developed specifically from a North American scholarship. While there is a good deal that these enterprises have in common, the US context should not be accepted uncritically as the basis for global studies of other systems.^25^ In summary, the anti-CRT movement is one of a number of simultaneous reactionary projects that are decades in the making.^26^

### Why CRT and what threats to the nation?

The anti-CRT campaign is simultaneously both nothing to do with, and definitely about CRT. On one level, using the term ‘CRT’ is a convenient device for invalidating any discussion of racism (as well as the connected ones on sexism, women’s reproductive health, sexuality etc.) by depicting it as un-American, shaming, unfair to children, unfair to white people, to men, to straight people, etc. On another level, concepts that are key to critical race scholarship, but which predate and exceed CRT, are part of what is targeted.

Originator of the anti-CRT campaign, Manhattan Institute political advisor Chris Rufo,^27^ appeared on the Tucker Carlson Show on Fox TV in September 2019. The Trump administration contacted him soon after, and an Executive Order was issued banning CRT from any federal training (Cineas, 2020). While this EO was immediately rescinded by President Biden on taking office, CRT had suddenly gained national ‘recognition’ after 30 years of being a relatively obscure body of ideas in a subfield of education, law and sociology (Meyer et al., 2021). However, this recognition came as a form of notoriety.

Rufo has labelled CRT ‘the perfect villain’, suggesting that the term be hammered home so that everyone associates it with something negative and ‘un-American’. He argues that CRT is divisive and racist (because it suggests one group is superior and one group is to blame, therefore making people responsible for, and guilty about the past). On his ‘briefing book website, Rufo states: ‘While most K-12 schools do not explicitly label their materials as “critical race theory,” any school that is teaching its core principles—such as whiteness, systemic racism, white privilege, and intersectionality—is, by definition, practicing critical race theory in the classroom’.^28^

These four key principles: whiteness, systemic racism, white privilege and intersectionality (I note that he omits ‘white supremacy’ from this list) are certainly operationalized in CRT, but have ideological lives that predate and exceed CRT, so what Rufo focuses on is casting the net widely enough to cover social science concepts that question and critique the racial order—from different angles. For Rufo, the narratives of CRT undermine those of America as a liberal democracy and meritocracy, by highlighting racialized inequality and continuities between slavery and post-slavery social orders. In summary, Rufo’s take on CRT is that it is racist because it identifies white people as the source of racism; divisive because it therefore creates distinctions among Americans; Marxist because some of it critiques capitalism; and is an ideology that in summary, ‘poisons the minds’ of young people. Rufo’s campaign strategy thus frames the battle as one over the future of the nation’s children, and incites activism from concerned parents to protect them from what is described as ‘political manipulation’. Identifying places where political pressure can be applied at School Board level, he has set out a blueprint for galvanizing ‘white innocence’ and middle-class white outrage. Moreover, concerned parents’ groups have successfully focused their anxiety and anger on a variety of targets within the equality, diversity and inclusion agenda in the 2022–24 period, such as gender identity, underscoring once more that the campaign against CRT cannot be fully understood as a stand-alone movement. It is one strand of the American counter-revolution.

The campaign links the republican base to State level legislators, granting the latter justification (if any were needed) to ‘respond’ to legitimate concerns’ about politicization of education. The outrage of parents (the majority of whom are White) is first focused on complaints to school boards. Currently this campaign mostly targets schools, but some States (e.g., Florida and Texas^29^) are also targeting higher education after successfully legislating against the teaching of race and gender in particular ways in K-12 education. For example, Florida’s House Bill 999 (March 2023) builds on the ‘Stop WOKE Bill’ from 2022, and seeks to remove ‘ideology’ and ‘indoctrination’ from public higher education by banning campus activities based on so-called ‘divisive’ concepts such as Intersectionality, CRT and Diversity, Equity and Inclusion. The Bill specifically prohibits ‘pedagogical methodology associated with Critical Theory, including, but not limited to, Critical Race Theory, Critical Race Studies, Critical Ethnic Studies, Radical Feminist Theory, Radical Gender Theory, Queer Theory, Critical Social Justice, or Intersectionality’.^30^ Moreover, the bill is not just about teaching, but about changing the status of tenure and conditions attached to it, to make it easier for faculty to be stripped of tenure and dismissed.

Section 3B of Texas HB3979 stipulates ten requirements and / or concepts that may not be used in a course, ranging from making individuals feel guilty about their history, to an argument that the institution of slavery was foundational to the USA, rather than an anomaly.^31^

The list of prohibited teaching provides a combination of wilful and politicised misinterpretations of the existing body of work on race and gender inequalities, and implicit refutations of ideas about white privilege, white supremacy and systemic racism that have trickled into school education from the social sciences in the last three decades. Item *x* is particularly interesting, given that all the social science concepts alluded to take as their departure points that sexism and racism are systemic, i.e., they are functions of the normal operation of the American social system, rather than anomalies.

The ‘authentic founding principles’ of the United States applied in the eighteenth century exclusively to the electorate of property-owning white men, evidencing the existence of a social hierarchy based on class, race, and gender. Yet even such a mundane affirmation of historical context would theoretically fall foul of HB 3979 article 3B (*x*). Quite apart from the authoritarian footprint involved in politicians monitoring university syllabi and interfering with academic freedom (Marlière, 2023), such Bills are attempts to suppress discussion of the existence and the validity of the racialized hierarchy, explore its history and raise awareness of white privilege, so that the long life of white supremacy becomes visible. What is novel in this period of counter acts is the contents of the discussion are explicitly spelled out in legislation that at State level is a set of variations on a theme.

There are multiple levels at which the ant-CRT campaign functions. One is the ideological and significant material basis supplied by the nexus of organisations such as the Heritage Foundation, the Idaho Freedom Foundation, the American Legislative Exchange Council (ALEC) that fund conservative political projects in the USA (Schwartz, 2021), such as Moms for Liberty (Little, 2021), and the media networks of Fox, Breitbart, Daily News, etc. (Gross, 2021), whose attention boosts membership and profile, Lastly, there are education-sector actors particularly, PragerU, the private-sector conservative education provider that creates anti-CRT material as well as its other resources for schools that states such as Florida have purchased in recent years (Archie, 2023). Another is the state-level legislation which is predominantly but not exclusively successful in Republican-run state legislatures. The last is the funded activism of groups following the blueprint of entryism to acquire control of school boards and then impose bans on course content.

### Anti-CRT work as ‘cement’ for populism

In the decades since the application of Nixon’s ‘Southern Strategy’ (Maxwell and Shields, 2019), Republicans have relied on white demographic (particularly with no college degree) to a far higher extent than Democrats.^32^ Trump’s vote relied on ‘white non-Hispanics’ in both 2016 (54% to the Democrats’ 39%) and 2020 (57 to 42%). Identifying concrete ideas that embody unwanted change sustains and / or activates people’s anxieties about losing ground materially and, in terms of identity, being a good person who holds American values that are under threat.

Opposed to these values are the so-called ‘identity politics’, the unravelling of American values (no abortion; individual freedoms) that are transgressed by collective rights, and evocations of links between the past and present that cannot stay buried. As the ‘good’ American values are understood to have been the core of society since the foundation of the nation (hence the references to it in TX HB 3979), the idea that even in the 1960s and 70s such values were overridden by racist practices spoils the narrative, which suggests that the 1965 Civil Rights Act was the cut-off point between the bad old days and the colorblind contemporary world.

So the anti-CRT campaign is not about the details of CRT *as such*, but about a much broader project, both in terms of anti-racism, and the multiple elements of progressive thought that are now under attack, with freedom of sexuality, access to reproductive rights, climate change, etc., as its targets. The anti-CRT campaign seeks to block discussion of racial inequalities, and to thwart and roll the spread of concepts drawn from social science analyses of racism back into the mainstream (white) understandings of the twenty-first century social order: in which white Americans are the real victims of racism. And to be clear, many of the concepts identified as ‘CRT’ (which dates back only to 1989) have (like structural / institutional / systemic racism) been live since the 1960s, predating CRT itself by a quarter of a century; ‘white privilege’ since the early 1980s, and ‘white supremacy’ since du Bois. Cramming every social science concept about race into the convenient toxic packaging of CRT enables the erasure of substantial historical detail and ideological sophistication.^33^

## Discussion: Making sense of counter acts

I have argued that the various campaigns run by the State(s) and social movements to challenge the basis of social science concepts that have been used as foundations for activism for the last few years in France and the USA, for example, should not be read solely as isolated cases of local struggles but also as strands of a ‘global pushback’ against antiracist ideas and practices, in which the nationalist political right and its networks in the media, government and funded civil society organisations are key players. The discourses driving the counter acts attempt to redraw national identity, depicting antiracist work (activism, scholarship, political practice) as a threat to the nation. Here I will explore those connections and parallels between France and USA. Focusing on content, engagement with individual nations’ values and with nationalist ideas.

The anti-antiracist political strategies outlined above have clear functions. They are a form of backlash characterising the populist authoritarian turn, in which ideological, legislative and cultural advances to disrupt the racial status quo—no matter how piecemeal, symbolic or ineffective—are represented as divisive threats to society whose agents are racialized minorities and their fellow travellers. The goal of the counter acts focused on here, which are about racialised inequalities, is to accomplish four things at once:

- maintain the racial status quo at the white supremacy setting^34^;
- sustain or expand the divisions in society, particularly by hailing white people as white, in order to defend ‘their’ interests.
- earn political capital for publicly defending the nation; and lastly,
- ensure the health of the project that has underwritten neoliberal governance since its implementation in the Transatlantic world in the early 1980s (the moment when, according to Ignazi, the populist right finds a wider, and more receptive audience for its message): the flow of power and resources from the majority to the wealthiest minority.

Although I will go onto to emphasize some connecting points, I would first stress the importance of putative national values in the argument over what is at stake, and what has been infringed—in the terms of these State counter acts. The key French value is secularity, the distinguishing concept and practice presented as governing the social relationships of its citizens in the French Republic. Secularity short-circuits ethnic and racial trajectories for valid resistance to racism, squeezing all struggles into a one-size-fits-all template that lacks specificity and is also incapable of critiquing itself, since the academic language and concepts that would enable that are marked as illegitimate (Kiwan et al., 2023). While the currently dominant version of secularity differs markedly from the original, this tells us at least as much, and possibly more about the populist right’s capacity for authoritarian reinterpretation than it does about the function of secularity. Via secularity, the French state has evolved ways to curtail free speech, cancelling particular concepts and invalidating the credibility of those who use them.

The American anti-CRT campaign has reached similar positions, albeit having travelled a different path. Although secularity (referred to as the separation of Church and State) is a foundation of the US constitution, it is not a useful tool in this American struggle. Considering the white conservative Christian demographic that is significant in the constituency mobilised by anti-CRT, it would be counter-productive. In fact, the values that the campaign is defending are not explicitly but implicitly marshalled. Sanctions apply to ideas that seek to indoctrinate children; divide Americans by race; make them feel responsible or guilty for the past; suggest that racial inequality is anything but an unwanted outcome rather than a deliberate objective of US systems of control; or to expose them to Marxism. Rufo’s playbook identifies these topics, which are clear from the wording of the various state government Bills. No-one, in the view of these legislators, is to be connected by pedagogy, either to the past or to society in any way that disturbs the status quo by encouraging critique. The anti-racist messengers who do exactly this are thus actually produced in this framing as the racists, breeding division into the cohesive Republic from within. The minimization and abstract liberalism strands of colorblind racism (Bonilla-Silva, 2021) explicitly underpin the project.^35^

The anti-CRT campaign seeks to demolish the fragile dominance of structural / systemic racism as a frame for understanding and explaining the intergenerational experiences of minorities and replace it with individual responsibility. We are stumbling here orthogonally into a national value that Americans consider more relevant than the separation of church and state: freedom of speech. The closing down of speech rights in this campaign functions via the threat of dismissal from teaching posts, not just in the K-12 system but also, where the state is particularly assiduous, e.g., Texas and Florida, in higher education. Yet anti-CRT’s capacity to override freedom of speech in such a peremptory way indicates the potency of the vision of national cohesion deployed as the utopia to the dystopia emerging from CRT’s critical framing.

### Counter revolutionary networks?

This article has identified multiple layers of activism entailed in producing counter acts. We have also seen that discourses about saving the nation from destruction are fundamental to the types of counter act analysed here. Here is a paradox: all this ideological labour is aimed at reimagining and purifying individual nations, yet the basis of this article is that beyond that pattern, there are (overlapping) networks of ideas and people in what thirty-two years ago for Ignazi (1992), was the space between the traditional, mainstream Right and Fascist Far-right, and whose objective is to create solidarity with like-minded activists and funders *from other nations*. I maintain that the anti-CRT campaign and the French state’s resistance to the concept of Islamophobia are part of the same global phenomenon of loosely orchestrated backlash. Steve Bannon’s story points to the limits of that orchestration. Bannon is an entrepreneur of radical populist nationalist ideas about saving Western civilisation from liberalism and its associated demons. He has attempted to work outside the USA since his period as Trump adviser (2016–17) and political commentator running the white nationalist Breitbart News. In 2018 he was brought in by Belgian People’s Party leader, Mischaël Modrikamen, to work from Brussels on ‘The Movement’, a pan-European project. His initial speech, in which he urged activists to wear accusations of racism as a ‘badge of honour’ (Willsher, 2018) garnered much media coverage. However, individual countries’ laws on electoral funding from foreign sources, and lukewarm responses from various parties (some of whose ideologies included anti-Americanism) reduced the impact he had sought, and hampered his ambition to be ‘the infrastructure, globally, for the global populist movement’ (Horowitz, 2018). Moreover, the 2019 European Parliamentary Elections that were the ostensible focus of that organisation’s work, yielded poorer results for the populists than had previous ones. Although Bannon was well-received in Brazil (Pagliarini, 2021), his actual contribution to Bolsonaro’s 2018 victory is unclear. However, he actively supported the theory that the 2022 election was stolen from Bolsonaro, and legitimized the attack on the congress after those elections (Palmer, 2023). Our learning is that first, the populist radical right nationalist world is nuanced: its European members are sensitive to American influence in politics. It is one of those ‘external threats’ that occupies a large place in its lexicon, even if US populists’ principles are close to identical with European ones. Even the powerful French anti-anti racists are wary of American influence. Second, the playbook in the Americas involves building expectations that democracy will be abused by the opponents, then encouraging anti-democratic direct action based on that expectation, with no evidence. This is an area of populist politics not replicated across the Atlantic, certainly deleterious to democratic processes, and which generates an increased risk of prosecution.^36^ As for Bannon’s plan for a global network of populist nationalism, ideas travel through this space more impactfully than people.

### Connecting the counter acts

In this brief summary I explicitly bring together the connections, noting the shared departure points for what are presented as exceptionalist stories in both France and the US. What is really exceptional are, (i) the timing and (ii), the specific context. Apart from that, their similarity is interesting. We should understand what the discourses and activities (especially those of the State) accomplish, as much as what they look like.

References to national values (secularity and meritocracy) are contrasted with those of the unpatriotic Left and politically-motivated minorities who lean on religious and/or racialized identities to do ‘identity politics’, and who point to inequalities rather than social mobility (Trump, 2020). Indeed, this reference to national values is in the circling-the-wagons-mode of beleaguered patriots, because that unpatriotic alliance is posited as being in the ascendancy. These anti-anti racist discourses only work through presenting a reversal of empirical power relations as the basis of their claims. Here, white conservatives are a beleaguered and oppressed group fighting back for the truth, and saving the nation from fifth columns.

These national values are centered on a particular universalist grasp of equality, as being a formal ideological property bestowed by the law, rather than the empirical practices that people experience. Colorblind racism (Carr, 1997; Bonilla-Silva, 2021) thus underpins both the US and France’s denial of the respective nations’ formative historical experiences (Beaman and Petts, 2020). It is the ideological refuelling point for both neo-secularity’s attack on the concept of Islamophobia and the anti-CRT campaign.

Colorblind racism always directs us to the ‘real’ racism that happened to other people, somewhere else, at some previous time. According to colorblind racism, what matters now is letting go of the (irrelevant) past, and focusing on the opportunities available in the republic. The State acts to guarantee freedoms in times of emergency, and in these times of ‘ideological emergency’, the state acts to restore balance in a playing field that was level but has now tilted too far towards the minorities.

The role of the State is to reframe the power relations through legislation that protects a particular, racialized understanding of American history and outlawing critical engagement with it. Legislation controlling religious organisations (France) and school curricula (USA) will thus protect the core of the nation and its essential values. We note the similarity of outcomes despite differences in national political structures: highly centralized v. highly decentralized governance, and in the principal targets of the counter act; African-Americans in the US, and Muslims in France.

French politicians engaged in the struggle against anti-racism often blame the US academy for divisive and foreign ideas that they claim are inappropriate for the French context, but the script they use is very similar to that of the American anti-CRT warriors. CRT and Islamophobia are both constructed as partisan Trojan Horses for transforming the Republic into a dystopia, whereas discourses supporting the contemporary unequal racial status quo are the neutral truth.

I have set ‘counter acts’ in a context of a global backlash against the BLM moment, and more broadly, against the advances made in civil society against structural and systemic racism in the last three or four decades. The counter acts aim to ‘change the narrative’ on racism, to reduce it to individual bad apples, to a relatively small issue that anti-racists and minority groups ‘exaggerate’ for political gain. Fighting the narrative that locates systemic racism within global systems and national histories, counter acts seek to decouple at least three pairs of elements:

- the State from a role in reproducing racism;
- the national story from the centrality of ongoing racism;
- and sociological theories from making sense of racism and therefore impacting resistance to it.

One tool for achieving these objectives is outrage, generated from the discrepancy between the perceived abstract ‘national values’ and those put forward as being the empirical national values by proponents of anti-racist scholarship and activism. The outrage is produced when people and / or concepts are identified as both invalid, and threats to the nation (i.e., the particular racialized order). Hence the effort engaged in via media sources in both sites to normalize the threat of Muslims and that of divisive ideas that imperil the nation.

Claims for justifying the suppression of discourse are based on two things: denial and the reversal of causation. First, the nation is proclaimed as a moral entity that has already achieved an admirable level of equality and democracy. France has procured a secular state with everyone equal in the eyes of the law as French citizens, while the USA’s American dream, where anyone can achieve anything with hard work is a potent myth with enough examples of success to sustain it, and provide a slice of exceptionalism.

Second, the relevant progressive movement is depicted as sowing division by focusing on inequality and using the concept of ‘race’. According to the logic of colorblind racism, the antiracists thus *cause* racism. At this point, the outrage derived from the ethical transgression of antiracist ideas means that the freedom of speech to which appeals are fundamentally made in this discourse no longer applies to the target ‘speech’ of antiracism. In other words, freedom of speech for the proponents of anti-Muslim racism in France and anti-CRT in the USA means being able to invalidate claims that the actions and ideas embodying racism (particularly at state level) are racist *at all*, and to claim victimhood instead: ‘those authoritarian liberals are preventing me exercising my 1^st^ amendment rights’. This is freedom of speech weaponized to protect the dominant groups’ ideas of social order rather than enabling the oppressed to speak back to power. This use of freedom of speech raises the question of distinct conceptions of the notion of freedom. For the many overlapping struggles to be portrayed by the more powerful as defending essential values leaves us with a stark discrepancy between freedom of speech conceived of as a means of ‘cancelling’ attempts to discuss US and French histories of power and social control, and freedom of speech understood as ‘freedom or death’ by minority groups resisting that social control, as Public Enemy (1989) assert.

We are in a specific phase of global white supremacy, in which its powers are simultaneously considerable and vulnerable—behind political losses as well as gains, and have pushed mainstream parties to adopt elements of its agenda. Considerable because the Right has effectively mobilized its cultural warriors to create contemporary ‘moral panics’ around fronts on which anti-racist activists (among others) are engaged, and has *de facto* networks of movements, plus parties that are in power, or power-adjacent, and therefore in a position to make political change. And vulnerable because of two things. First, in explicitly identifying these areas as sites of cultural struggle (around the meanings and politics of ideas), these counter acts engage new actors in new ways and cannot exert lasting control over the readings of these acts. Such forms of politics produce antiracists as well as white supremacists. Second, the fact that theories and practices of whiteness are made explicit reveals a degree of progress in engaging it that has forced groups concerned by a perceived threat to white supremacy to enter a battlefield at all: white supremacy no longer goes without saying. Power is no longer entirely invisible, and what is visible is easier to critique and engage with than what is invisible.

Campaigns and strategies aiming to invalidate and discredit social science concepts that help us to understand racism require an extraordinary sleight of hand deployed to decisively ‘change the narrative’—away from systems of oppression to individual choices; and posit national cohesion as constructed in a way that definitively closes down the possibility of understanding the oppression of groups whose citizenship is already tenuously held. Critiques of inequality *per se* are thus constructed above all as anti-patriotic, which is typical of authoritarian regimes. The question that arises from the Right’s extended foray into wars of definition is, are we now witnessing the revival, the hegemonic peak, or the ultimate dying backlash of white supremacy?

## Data availability statement

The original contributions presented in the study are included in the article/supplementary material, further inquiries can be directed to the corresponding author.

## Author contributions

SG: Writing – original draft, Writing – review & editing.

## Footnotes

^1^While this is a crucial context, it has to be omitted in this article, where the focus is on the role of the (central and regional) state.

^2^In some respects, this populist discourse has barely changed since the 1980s. In the UK, the right populist ‘National Conservatives’ held their first conference in 2023. Conservative MP Miriam Cates states that, “national conservatism” developed from the 2016 Brexit referendum and Tory 2019 election victory. Those events, she maintains, were an “instruction from the public that they expect us to govern with their interests, their values in mind. Not the values of the intelligentsia – the globalised elite whose loyalties are to everyone and no one” (Adu and Walker, 2023). This classic populist refrain frequently articulated by members of the establishment elite trying to designate other members of the elite as more and differently elite than them. Cates’ comments could have come from a right populist fringe politician in the early 1980s. However, none of those politicians would have been serving members of a mainstream party holding a parliamentary majority, which is why this example bears out the basis of my scenario.

^3^ https://nationalconservatism.org/

^4^‘The State’ here includes state government in the USA, which has an important degree of autonomy when it comes to legislative functions and potential control of education.

^5^*Cf* Former Home Secretary, Theresa May’s claim that ‘inequality is a dirty word’ (May, 2010).

^6^I translated the English version of the book by Abdellali Hajjat and Marwan Mohammed, *Islamophobie: comment les élites françaises construisent le problème musulman*, on which I draw heavily. The English version is *Islamophobia in France*, University of Georgia Press, 2013.

^7^Indeed, I am a co-author of a book in which ‘the global racialization of Muslims’ is preferred, with an explanation of the choice of words (Selod et al., 2024).

^8^La France est. une République indivisible, laïque, démocratique et sociale. Elle assure l’égalité devant la loi de tous les citoyens sans distinction d’origine, de race ou de religion.

^9^Elle respecte toutes les croyances.

^10^Bravo López (2011) identifies the first use of the term ‘islamophobie’ in French in the work on colonial administrators in the French colonies of West Africa in the early 20th century. They also argue that there is no translation of Islamophobia in either Farsi or Arabic, making it impossible for the term to have been used systematically against opponents of the Iranian Islamic regime that overthrew the Shah in 1979.

^11^Baroin is a centre-right politician who was in Jacques Chirac’s Union for a Popular Movement (UMP) and then the Republican party (and briefly Finance Minister 2011–12). Prime Minister Jean-Pierre Raffarin commissioned a report on French nationality and Islam derived from a series of round tables held in 2003. The original text can be accessed at: https://www.voltairenet.org/rubrique506.html?lang=fr.

^12^The French press law of 1881 included the offence of “apology for terrorism.” The November 2014 law transferred it to the French Penal Code. Since then, the State has used Penal Code Article 421-2-5, which sets out punishments of up to five years in prison and a fine of up to €75,000. Online offenses can be punished with seven years in prison and a €100,000 fine.

^13^In 2017, Education minister, Jean-Michel Blanquer, sued the trade union SUD for holding a workshop on state racism at its annual congress.

^14^He received 7.1% of the vote in the first round of voting in April 2022, and went on to endorse Le Pen’s run for the second round, where she received 41.5% of the vote (Voce and Clarke, 2022).

^15^ https://www.mediapart.fr/

^16^As opposed to elsewhere in the world, which is the usual profile of the experts used by the French state, who actually have very little professional expertise on contemporary Islam in France.

^17^Loi confortant les principes républicains et de lutte contre le séparatisme.

^18^Wolfreys (2023, 170–171) reports that: ‘By October 2020, over 200 drinking establishments had been shut down, along with 15 places of worship, four schools and 13 associations or cultural centres (Macron 2020). During the course of 2021, 2,623 prayer rooms and mosques were ‘screened’ on instruction from the interior ministry (*Le Monde*, 11 January 2022)… By early 2022, nearly 25,000 premises had been put under investigation, with more than 700 closed and 46 million euros seized’.

^19^The French text of the Charte de Respect des Valeurs de la République et du Principe de Laïcité can be found at: https://en-marche.fr/charte.pdf.

^20^*Le Point*, 2 December 2020: Le Collectif contre l’islamophobie en France officiellement dissous: https://www.lepoint.fr/societe/le-collectif-contre-l-islamophobie-en-france-officiellement-dissous-02-12-2020-2403864_23.php. Depuis plusieurs années, le CCIF conduit avec constance une action de propagande islamiste; *Radio France*, 19 October 2020: Qu’est-ce que le CCIF, l’association que Gérald Darmanin veut dissoudre? https://www.radiofrance.fr/franceinter/qu-est-ce-que-le-ccif-l-association-que-gerald-darmanin-veut-dissoudre-7700022—“C’est. une association qui touche des aides d’État, des déductions fiscales. Il y a un certain nombre d’éléments qui nous permettent de penser que c’est. un ennemi de la République.”

^21^Whether this is a distortion of the original aims of laïcité, or merely a transformation of an elastic term, it is clear that the latest iteration of secularity revolves around the ‘Muslim problem’ as a binary foil to Republican values.

^22^https://worldpopulationreview.com/state-rankings/states-that-have-banned-critical-race-theory (MAP) Details of existing Bills and Laws. Ed Week: https://www.edweek.org/policy-politics/map-where-critical-race-theory-is-under-attack/2021/06; See also Cornell Law Professor William Jacobson’s website, which reports supportively on the advance of anti-CRT initiatives: https://criticalrace.org/.

^23^Including the American Civil Rights Institute, Center for Equal Opportunity, Center for Individual Rights, Institute for Justice, and the Civil Rights Practice Groups of the Federalist Society (Embrick et al., 2020: 217).

^24^‘We define this whitelash as an individual, institutional, and/or structural countermeasures against the dismantling of white supremacy or actions, real or imagined, that seek to remedy existing racial inequities. Whitelash, we argue, is a reaction to challenges made to the white status quo; it is a reaction to growing diversity; it is a reaction against progressive changes (perceived or real) that would call out racism, question white privilege, or suggest racial equality is necessary to meet American ideals of fairness, in many of its forms’ (Embrick et al., 2020: 203).

^25^And I am aware that this wariness toward American influence may be construed as mirroring that of the populist nationalist groups I am writing about.

^26^It’s the focus because this article is on concepts that involve linking the nation with race. Another one on DEI is under construction.

^27^Rufo is a key actor in the contemporary national conservative movement, prominent also in the campaign to have the Presidents of Harvard, Penn State and MIT removed, ostensibly over anti-semitism in late 2023-early 2024. The presence of a network of organisations working on conservative projects is referred to below, but that network is the subject of a piece of work currently in development.

^28^https://christopherrufo.com/crt-briefing-book/ K-12 refers to the education of children from Kindergarten to 12th grade (age 17–18).

^29^While I was writing this paper, the Supreme Court ruling on Affirmative Action occurred. In the consequent SB17, the State of Texas quickly banned universities from hiring based on race and also from engaging in diversity, equality and inclusion activities.

^30^Section 3c of Florida HB 999 states: ‘General education core courses may not distort significant historical events with misleading or incorrect presentations of fact and must not include curriculum that is based on or otherwise utilizes pedagogical methodology associated with Critical Theory, including, but not limited to, Critical Race Theory, Critical Race Studies, Critical Ethnic Studies, Radical Feminist Theory, Radical Gender Theory, Queer Theory, Critical Social Justice, or Intersectionality, as defined in rules and regulations of the State Board of Education and the Board of Governors, respectively’.

^31^‘A teacher … may not (B): require or make part of a course the concept that: (i) one race or sex is inherently superior to the other; (ii) an individual, by virtue of the individual ‘s race or sex, is inherently racist, sexist, or oppressive, whether consciously or unconsciously; (iii) an individual should be discriminated against or receive adverse treatment solely or partly because of the individual ‘s race; (iv) members of one race or sex cannot and should not attempt to treat others without respect to race or sex; (v) an individual ‘s moral character, standing, or worth is necessarily determined by the individual’s race or sex; (vi) an individual, by virtue of the individual ‘s race or sex, bears responsibility for actions committed in the past by other members of the same race or sex; (vii) an individual should feel discomfort, guilt, anguish, or any other form of psychological distress on account of the individual ‘s race or sex; (viii) meritocracy or traits such as a hard work ethic are racist or sexist or were created by members of a particular race to oppress members of another race; (ix) the advent of slavery in the territory that is now the United States constituted the true founding of the United States; or (x) with respect to their relationship to American values, slavery and racism are anything other than deviations from, betrayals of, or failures to live up to, the authentic founding principles of the United States, which include liberty and equality’.

^32^Nevertheless, we note that there is a trend in conservative Latinx republican voting in both TX and CA for example.

^33^Actual CRT is an important arsenal but it’s not the only game in town. Indeed, it overlaps with critical race studies and critical whiteness studies, for example.

^34^White supremacy in the CRT sense of the term, as a system set up to benefit white people over other groups, ‘a political, economic, and cultural system in which whites overwhelmingly control power and material resources, conscious and unconscious ideas of white superiority and entitlement are widespread, and relations of white dominance and non-white subordination are daily re-enacted across a broad array of institutions and social settings’ (Ansley, 1997: 592).

^35^The four central frameworks of color-blind ideology are abstract liberalism (explaining racial matters in an abstract, decontextualized manner), naturalization (naturalizing racialized outcomes such as neighborhood segregation), cultural racism (attributing racial differences to cultural practices), and minimization of racism (downplays its frequency, extent and impact).

^36^Indeed, Bannon is currently appealing a four-year prison sentence for two charges contempt of Congress after the Jan 6 investigations, as well as under investigation for fraud in relation to the ‘We Build the Wall’ crowdfunding project.
